# Inhibition of Rho and Rac Geranylgeranylation by Atorvastatin Is Critical for Preservation of Endothelial Junction Integrity

**DOI:** 10.1371/journal.pone.0059233

**Published:** 2013-03-13

**Authors:** Hongbing Xiao, Xiong Qin, Ding Ping, Keqiang Zuo

**Affiliations:** 1 Department of General Surgery, Shanghai Tenth People’s Hospital, Tongji University, Shanghai, China; 2 Department of Cardiothoracic Surgery, Shanghai Tenth People’s Hospital, Tongji University, Shanghai, China; Sudbury Regional Hospital, Canada

## Abstract

**Background:**

Small GTPases (guanosine triphosphate, GTP) are involved in many critical cellular processes, including inflammation, proliferation, and migration. GTP loading and isoprenylation are two important post-translational modifications of small GTPases, and are critical for their normal function. In this study, we investigated the role of post-translational modifications of small GTPases in regulating endothelial cell inflammatory responses and junctional integrity.

**Methods and Results:**

Confluent human umbilical vein endothelial cell (HUVECs ) treated with atorvastatin demonstrated significantly decreased lipopolysaccharide (LPS)-mediated IL-6 and IL-8 generation. The inhibitory effect of atorvastatin (Atorva) was attenuated by co-treatment with 100 µM mevalonate (MVA) or 10 µM geranylgeranyl pyrophosphate (GGPP), but not by 10 µM farnesyl pyrophosphate (FPP). Atorvastatin treatment of HUVECs produced a time-dependent increase in GTP loading of all Rho GTPases, and induced the translocation of small Rho GTPases from the cellular membrane to the cytosol, which was reversed by 100 µM MVA and 10 µM GGPP, but not by 10 µM FPP. Atorvastatin significantly attenuated thrombin-induced HUVECs permeability, increased VE-cadherin targeting to cell junctions, and preserved junction integrity. These effects were partially reversed by GGPP but not by FPP, indicating that geranylgeranylation of small GTPases plays a major role in regulating endothelial junction integrity. Silencing of small GTPases showed that Rho and Rac, but not Cdc42, play central role in HUVECs junction integrity.

**Conclusions:**

In conclusion, our studies show that post-translational modification of small GTPases plays a vital role in regulating endothelial inflammatory response and endothelial junction integrity. Atorvastatin increased GTP loading and inhibited isoprenylation of small GTPases, accompanied by reduced inflammatory response and preserved cellular junction integrity.

## Introduction

Statins, a family of 3-hydroxy-3-methylglutaryl coenzyme A reductase (HMG-CoA reductase) inhibitors, have been used extensively to block cholesterol biosynthesis and reduce serum cholesterol. Recent evidence shows that statins have pleiotropic effects, such as inhibition of pulmonary hypertension and attenuation of β-amyloid-induced microglial inflammatory response in Alzheimer’s disease patients that are distinct from their cholesterol-lowering actions [1]. Statin treatment in mice enhanced endothelium-dependent relaxation to acetylcholine, whereas statin withdrawal elicited oxidative stress and attenuated endothelium-dependent relaxation, suggesting that statins directly affect endothelial cell function [2]. Key to our current study, statins treatment produced an outstanding enhancement of endothelial barrier function, including inhibition of stress fiber formation induced by various factors [3], [4]. These beneficial effects clearly show that statins have a fundamental mechanistic effect on the endothelium, independent from inhibiting cholesterol synthesis [5].

Small GTPases, including Rho, Rac and Cdc42 are central to controlling cytoskeletal rearrangement [6]–[8]. Small GTPases can activate myosin light chain kinase (MLCK), and the phosphorylation of myosin light chain by activated MLCK leads to cytoskeletal rearrangement, including cellular constriction or relaxation [9]. This pathway accompanies changes in cellular junction proteins and endothelial barrier function, which are critical for many processes, including neutrophil and macrophage migration, lamellipodia formation, and regulation of endothelial barrier integrity.

Two key post-translational modifications regulate small GTPase protein function: exchange of bound GDP for GTP and lipidation. Exchange of GDP for GTP converts the inactive GDP-bound GTPase to the active GTP-bound form [10], [11]. Conversion of the GTPase to the active GTP-bound form is termed GTP loading. Lipidation involves modification of small GTPases with either geranylgeranyl pyrophosphate (GGPP) or farnesyl pyrophosphate (FPP), and is required for targeting small GTPases to the cell membrane, although the functional consequences of GTPase lipidation are not fully understood [12]. In this process, small GTPase proteins are covalently attached to GGPP (for Rac, Rho and Cdc42) or FPP ( for Ras), primarily at C-terminal cysteine residues, to become lipid avid [13], [14]. Since GGPP and FPP are are down-stream products of HMG-CoA reductase, lipidation is also blocked by statins [15].

Interestingly, statins appear to be involved in both GTP loading and lipidation of small GTPases. Statins have been reported to increase Rac-GTP loading accompanying a decrease of Rho-GTP loading [16], which may contribute to the endothelial barrier enhancement effects accompanying junction formation [17]. The functional significance of inhibiting small GTPase lipidation using statins has not been well characterized in endothelial cells. Simvastatin interferes with angiogenesis by inhibiting geranylgeranylation of Rho [18], and also abolished VEGF-mediated inside-out signaling via inhibition of Rho-GTP [19], while lovastatin inhibits epithelial stress fibers accompanying the loss of focal adhesions by impairing Rho and Rac GTPase geranylgeranylation. However, the importance of the relationship between GTP-loading and post-translational lipidation of small-GTPase proteins remains undefined.

In the present study, we focused on the role of lipidation in regulating small GTPase functions involved in endothelial inflammatory processes and cytoskeletal structure. We showed that atorvastatin inhibited endothelial cellular inflammation and cytoskeletal rearrangement by inhibiting small GTPase geranylgeranylation, which was reversed by exogenous geranylgeranyl pyrophosphate. We further identified Rho and Rac as the critical small GTPases responsible for the atorvastatin-mediated inhibition of endothelial inflammatory responses and cytoskeletal rearrangement.

## Materials and Methods

### Materials

Human Rac, Rho and Cdc42 antibody were purchased from Upstate (Charlottesville, VA). Rac, Rho and Cdc42 GTPase activity assay kits were also purchased from Upstate. The FITC-Dextran permeability kit was purchased from Millipore (Billerica, MA). GGTI-2133, FTI-276 trifluoroacetate salt and other chemicals were purchased from Sigma (St. Louis, MO). IL-6 and IL-8 cytokine kits were purchased from Invitrogen (Carlsbad, CA). The ECIS was purchased from Applied Biophysics Inc, (Troy, NY). Human Rho, Rac and Cdc42 siRNA (SMART pool) were purchased from Dharmacon (Chicago, IL). Human umbilical vein endothelial cells (HUVEC) were purchased from Millipore, and were cultured in complete EGM-2 medium (Lonza, Walkersville, MD) supplemented with 2% FBS.

### Membrane preparation and solubilization

80% confluent HUVECs were treated with atorvastatin in the presence of MVA, FPP, or GGPP as indicated, washed with ice-cold PBS, and harvested with hypotonic buffer (5 mM Tris-HCl, pH 7.6, 2 mM EDTA, containing protease inhibitors, 1mM NaVO4 and 20 mM NaF). The detached cells were homogenized with a pellet homogenizer twice on ice. The homogenate was then centrifuged at 400×g for 10 min at 4°C, and the supernatant retained and centrifuged at 12000×g for 30 min. The resulting pellet was washed three times with PBS containing protease inhibitors, solubilized in HEPES buffer containing 1% Triton-X100 for 1 hour on ice, then centrifuged at 14000×g for 30 min at 4°C. The resulting supernatant was collected for protein assay or small GTPase activity assay [20].

### Cytokine measurement

HUVECs were treated with atorvastatin overnight in the presence of MVA (100 µM), FPP (10 µM) or GGPP (10 µM), then challenged with LPS (100 ng/mL) for 6 hours. The medium was then collected by centrifuging at 500×g for 15 min. IL-6 and IL-8 in the medium was quantified using commercial kits from Invitrogen (Carlsbad, CA).

### Western Blotting and small GTPase activity (small GTPase GTP-loading)assay

Equal amounts of protein from cell membrane or cytosolic fractions, or from whole cell lysates, were mixed with Laemmli sample buffer and subjected to electrophoresis on SDS-PAGE gels, followed by transfer to nitrocellulose membranes (Bio-Rad Laboratories, Hercules, CA). After blocking, the transfer membranes were incubated with the specified primary antibody, followed by the appropriate HRP-conjugated secondary antibody. The immunoreactive protein content on the membranes was quantified by chemiluminescence (Amersham Biosciences, Piscataway, NJ) using Alpha Imager software (Alpha Innotech, San Leandro, CA).

Small GTPase GTP-loading assays were performed using commercial assay kits from Upstate Biotechnology. Briefly, the cell lysates were collected after treating HUVECs with agents of interest. The GTP-bound small GTPases were captured by immobilized PBD domain (Rac and Cdc42) or RDB domain (Rho). After washing the beads three times, GTP-bound small GTPases were released by boiling with Laemmli sample buffer. Following standard western blot protocols, the GTP content in small GTPases were determined as the amount of Rac, Rho or Cdc42 pulled down [21]. GTP-loading was expressed as the amount of GTP-bound small GTPase relative to total GTPase at each timepoint, and then normalized to the value at the zero hour timepoint.

### Measurement of transendothelial electrical resistance (TER) using ECIS

Measurement of transendothelial electrical resistance (TER) across confluent HUVEC monolayers treated with atorvastatin were performed using electrical cell-substrate impedance sensing system (ECIS, Applied Biophysics, Troy, NY) as described by Birukova AA et al [21]. Data pooling and analysis were performed using Epool software created in house and expressed either as normalized resistance relative to the point of agonist challenge. TER values from multiple independent experiments corresponding to each experimental condition were pooled at different time points and plotted versus time as the means ± S.E.

### *In vitro* vascular permeability measurement

A commercially available kit (Chemicon, Temecula, CA) was used for HUVEC monolayer permeability measurement. Briefly, HUVECs were seeded in the kit-supplied transwell insert and, after attaining confluence, were challenged with thrombin (1 U/ml) under various conditions as indicated. 2000kD FITC-dextran was then added into the insert. After 1 hour incubation with FITC-dextran, the insert was removed and the medium in the bottom chamber were collected. FITC-dextran concentration in the bottom chamber medium was assayed by fluorescent density, using a Titertek Fluoroskan II Microplate Fluorometer (Diversified Equipment, Lorton, VA) at excitation and emission wavelength of 485 and 530 nm, respectively [22].

### Endothelial cell immunocytochemistry

HUVECs were grown on gelatinized cover slips prior to atorvastatin treatment, followed by various stimuli as described. HUVECs were then fixed in 3.7% formaldehyde and permeabilized with 0.1% TritionX-100 for 10 min. The slides were washed in phosphate-buffer saline, blocked with 1% bovine serum albumin in phosphate-buffer saline for 1 hour, and then incubated for 1 hour at room temperature with primary antibody. After washing, the slides were incubated with dye-conjugated secondary antibody for 1 hour at room temperature, along with Texas-red-conjugated phalloidin for F-actin staining. After further washing with phosphate-buffer saline, cover slides were mounted using slow fade mount solution (Molecular Probes, Inc., Eugene, OR) and analyzed using a Nikon Eclipse TE 300 microscope equipped with a Sony Digital Photo camera DKC 5000. Images were recorded and saved using Adobe Photoshop [21].

### Statistical Analysis

Student’s t-test was used to compare the means of data from two different experimental groups, whereas significant differences –among multiple groups comparison (*P*<0.05) were determined by one way ANOVA and Tukey’s studentized range test. The results were represented as the mean ± S.E.

## Results

### Small GTPase geranylgeranylation mediates inflammation in LPS challenged HUVECs

**Figure 1:**
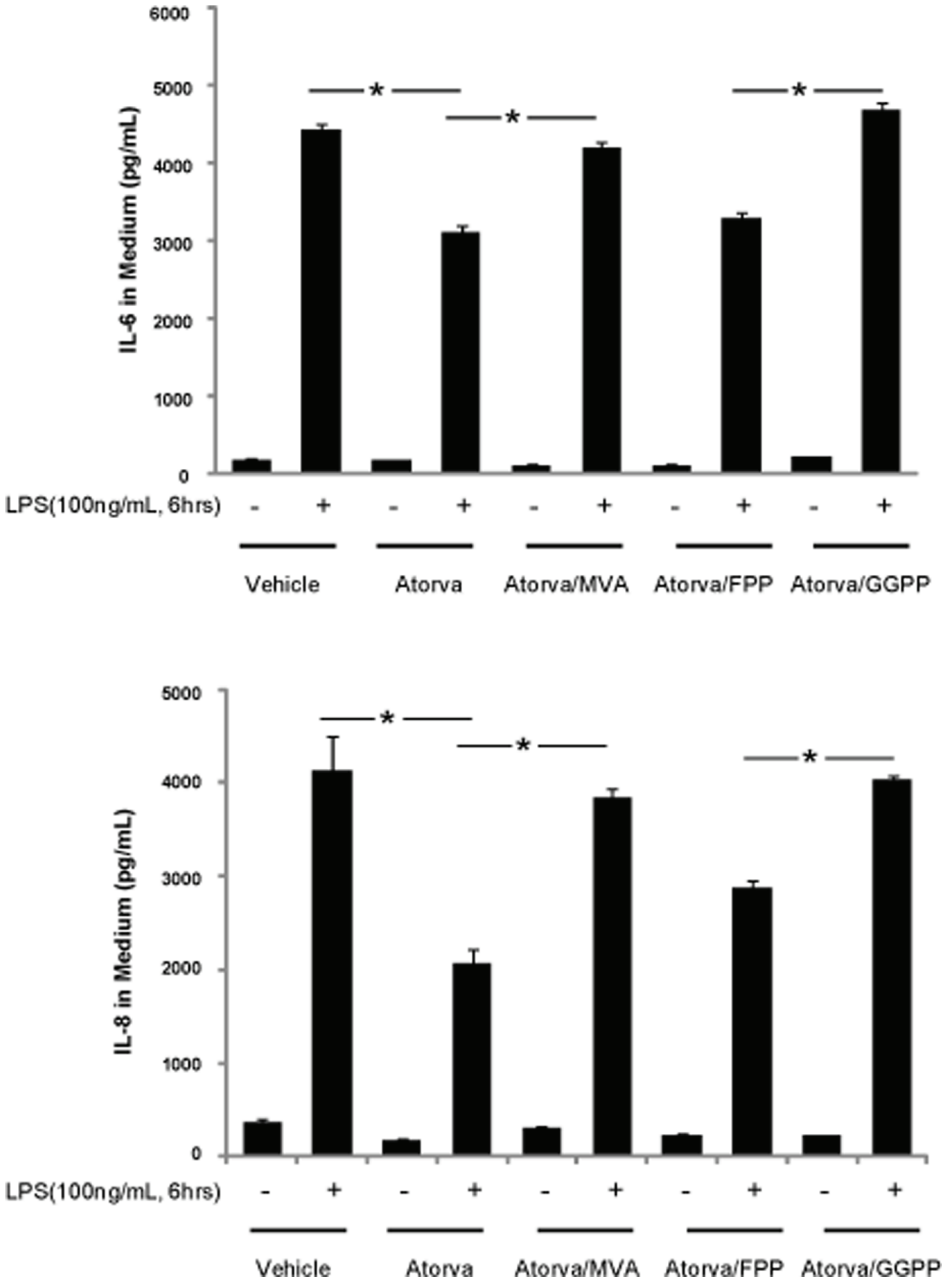
Atorvastatin inhibits LPS-induced inflammatory response in HUVECs. HUVECs grown to 80% confluence were treated with atorvastatin (10 µM) alone, or in the presence of either MVA (100 µM), FPP (10 µM) or GGPP (10 µM) overnight, followed by 100 ng/mLLPS for 6 hours. The medium was harvested, and IL-6 and IL-8 concentrations determined by ELISA. Data are expressed as means ± S.E. (*n* = 6 samples for control group; n = 8 for LPS treatment group), * *p*<0.05.

Small GTPases play a vital role in various cellular functions, such as survival, migration, junction formation, and inflammation. While numerous studies have shown that GTP loading is critical for small GTPase function, the relative importance of small GTPase binding with the cell membrane is still unclear. As shown in figure 1, atorvastatin, inhibits IL-6 and IL-8 generation induced by LPS. Compared to the LPS challenged controls (4300±230 pg/mL and 4100±630 pg/mL IL-6 and IL-8 ,respectively), IL-6 and IL-8 generation are significantly decreased in LPS challenged cells pretreated with atorvastatin (2950±120 pg/mL and 2100±420 pg/mL IL-6 and IL-8, respectively). MVA, an intermediate of cholesterol synthesis downstream of HMG-CoA synthase, reversed the anti-inflammatory effect of atorvastatin. IL-6 and IL-8 production in HUVECs challenged with LPS after treatment with atorvastatin and MVA were 4800±1200 pg/mL and 3800±1400 pg/mL, respectively. Interestingly, geranylgeranyl pyrophosphate (GGPP), but not farnesyl pyrophosphate (FPP), attenuated the anti-inflammatory effect of atorvastatin. IL-6 and IL-8 production in LPS-challenged HUVECs treated with atorvastatin and FPP was 3500±130 pg/mL and 2800±1470 pg/mL, respectively, compared to 5050±230 pg/mL and 4020±580 pg/mL IL-6 and IL-8, respectively, in HUVECs treated with atorvastatin and GGPP. These results indicate that small Rho GTPases, rather than Ras GTPases (FPP is required for Ras farnesylation and translocation of Ras to the cellular membrane), mediate atorvastatin’s anti-inflammatory effects.

### Atorvastatin increased GTP loading and translocation of small GTPases from membrane to cytosolic fractions

**Figure 2:**
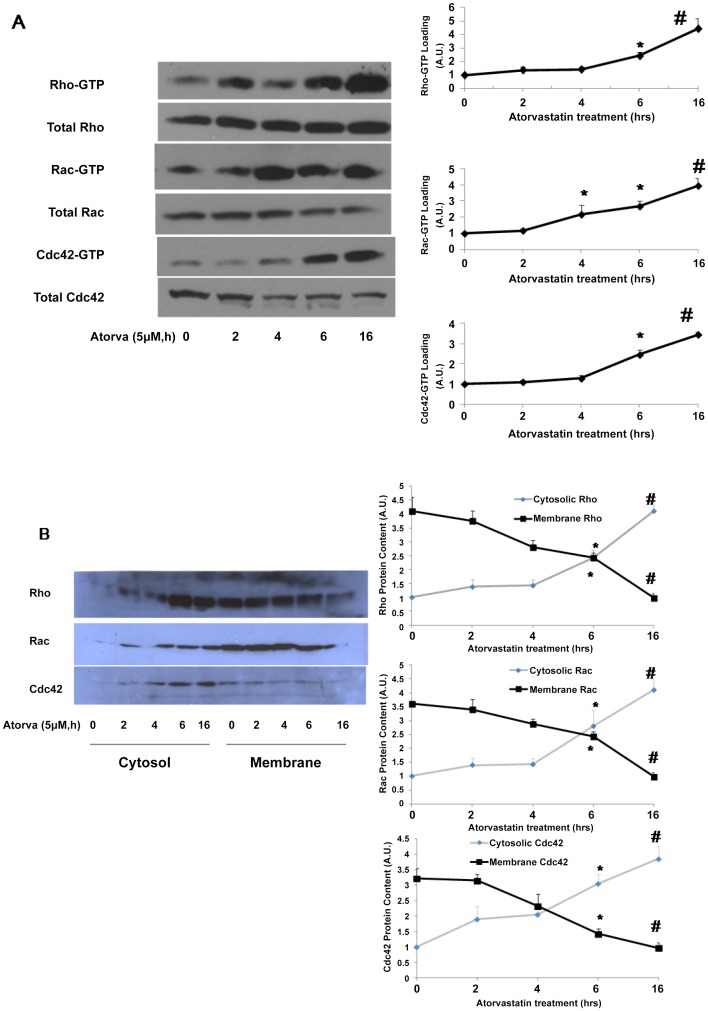
Atorvastatin treatment increased GTP loading and translocation of small GTPases from cell membrane to cytosol. (A) HUVECs were treated with atorvastatin (10 µM) for the duration indicated. The whole cell lysates were harvested, and Rho, Rac and Cdc 42 GTP-loading assayed as described in Methods. (B) HUVECs were treated with atorvastatin (10 µM) overnight, the cells collected, and the small-GTPase GTP-loading of cytosol and cell membranes fractions prepared as described in Methods. Equal amounts of cell membrane and cytosolic protein were suspended in Laemmli buffer, and the GTPase loading determined by Western blotting using Rho, Rac and Cdc42 antibodies. Data in shown in graphs represent the means ± S.E. for 4 samples at each timepoint, * *p*<0.05 vs control; #P<0.01 vs control.

The effect of atorvastatin treatment of HUVECs on GTP loading, an important regulator of GTPase function, was determined for Rho, Rac and cdc42 . As shown in figure 2A (top panel), Rho GTP loading increased after atorvastatin treatment to approximately 5-fold that of controls at 16 hours. Atorvastatin treatment also increased Cdc42 and Rac GTP loading in a similar pattern as Rho (figure 2 A, middle and bottom panels). Notably, atorvastatin treatment did not change total Rho, Rac and cdc42 protein in HUVECs.

Since statins inhibit synthesis of FPP and GGPP, which are required for small GTPase lipidation, we next investigated the effects of atorvastatin on the targeting of small GTPases., Atorvastatin treatment caused a time-dependent increase in Rho in the cytosolic fraction of HUVECs, accompanied by a time-dependent decrease of Rho density in cellular membrane (figure 2 B, top panel), reaching a maximum effect at 16 hours. Similar effects were observed on Rac and cdc42, though the timing may be different (figure 2 B, middle and bottom panels), finding which are consistent with previous reports [3].

Our findings raise an interesting question, which will be addressed in future studies, regarding the relative importance of GTP loading versus isoprenylation to the regulation of small GTPases in their role as mediators of multiple endothelial cell processes.

### Atorvastatin-mediated translocation of small GTPases is reversed by exogenous GGPP

**Figure 3:**
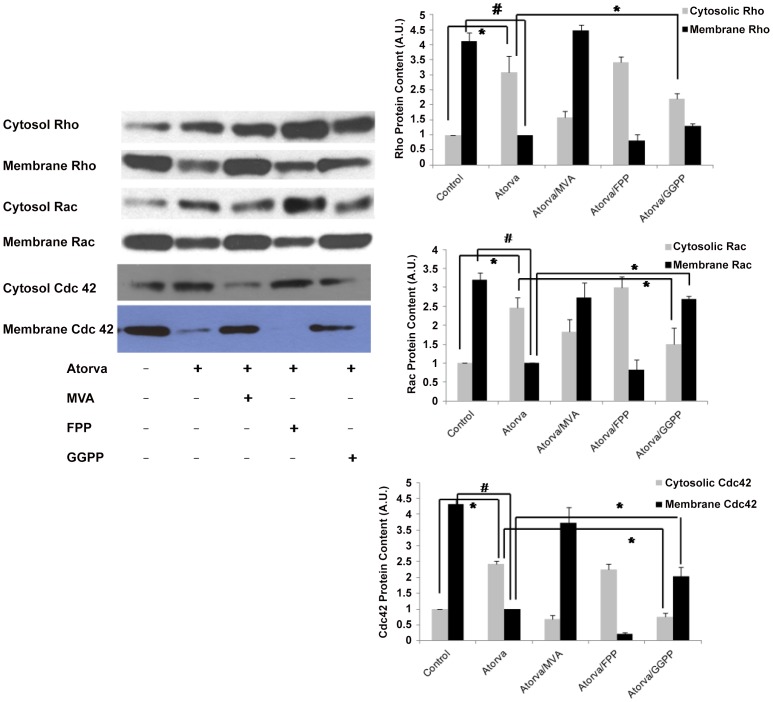
Exogenous GGPP, but not FPP, reverses atorvastatin-mediated small GTPase translocation. The Rho GTPases, including Rac1, RhoA and Cdc42 demonstrate translocation from the EC membrane to the cytosol in response to atorvastatin (5 µM, 16 h). This effect is consistent with inactivation of Rho GTPases at the cell membrane by atorvastatin, and is inhibited by MVA (100 µM, 16 h) and GGPP (10 µM, 16 h) but not FFP (10 µM, 16 h) implicating effects specific to geranylgeranylation inhibition. Data in shown in graphs represent the means ± S.E. for 4 samples for each treatment. Statistically significant differences between selected treatment groups were highlighted, * *p*<0.05; #P<0.01.

To further confirm the importance of isoprenylation to small GTPase function, we measured Rho, Rac and Cdc42 content in cellular membrane and cytosolic fractions after various treatments. As shown in figures 2 B and 3, atorvastatin decreased Rho content in cellular membrane and increased cytosolic Rho content. This atorvastatin-mediated Rho translocation was reversed by addition of 100 µM MVA and 10 µM GGPP, but not by10 µM FPP (Figure 3, top). Similar to Rho, atorvastatin also caused the translocation of Rac and Cdc42 to the cytosol from the cell membrane, which was also reversed by 100 µM MVA and 10 µM GGPP, but not by10 µM FPP. These finding indicate that geranylgeranylation, but not farnesylation, of these small GTPases is required for their binding to cellular membrane.

### Small GTPase geranylgeranylation regulates HUVEC monolayer permeability

**Figure 4:**
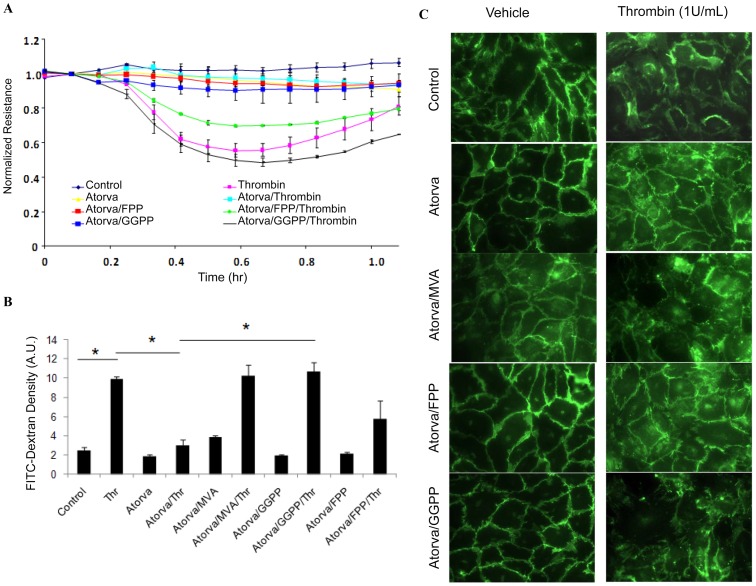
Atorvastatin preserved endothelial barrier integrity. (A) atorvastatin enhances endothelial cell junctional integrity. Changes in transendothelial electrical resistance (TER) were recorded by ECIS under varying conditions. Thrombin treatment caused a dramatic decrease in resistance, reaching a maximum at 30 min (40%), reflecting decreased junction integrity. Note that TER is preserved by treatment with atorvastatin prior to thrombin challenge, while co-treatment with GGPP (10 µM) but not by FPP (10 µM), attenuated the effect of atorvastatin. (B) HUVECs junction integrity was assessed by transwell permeability assay. Thrombin (1 U/mL) challenge caused a dramatic increase in HUVEC monolayer permeability, which was preserved by pretreatment with atorvastatin (10 µM). Similar to our ECIS results, atorvastatin-mediated preservation of cell junction integrity was reversed by MVA (100 mM, 16 h) and GGPP (10 mM, 16 h), but not FFP (10 mM, 16 h), * *p*<0.05. (C) Immunocytochemistry showed that atorvastatin pretreatment preserved VE-cadherin junctions against thrombin-challenge. Co-treatment with MVA (100 mM, 16 h) and GGPP (10 mM, 16 h), but not FFP (10 mM, 16 h) attenuated the protective effect of atorvastatin on VE-cadherin junctions.

Endothelial junction integrity is a critical aspect of many cellular processes, including inflammation and edema. To investigate the importance of GTPase isoprenylation to endothelial junction integrity, we measured the transendothelial electrical resistance (TER) of HUVEC monolayers, using ECIS (see “Materials and Methods”), under varying conditions. Compared to untreated controls, thrombin challenge caused a significant 45% decrease in HUVEC monolayer resistance, reflecting decreased monolayer integrity and increased permeability (figure 4A). Atorvastatin pretreatment significantly preserved HUVEC monolayer resistance after thrombin challenge, whereas co-pretreatment with 10 µM GGPP, and to a lesser extent with 10 µM FPP, reversed the protective effect of atorvastatin.

To confirm our observations using TER, we next used a transwell permeability assay (see “Materials and Methods”) to assess endothelial barrier integrity in HUVECs pretreated with atorvastatin alone or in combination with exogenous GGPP or MVA. As shown in figure 4B, challenge with 1U/ml thrombin significantly increased FITC-dextran leakage from insert into the transwell bottom chamber relative to controls (2.5±0.36 vs. 9.94±0.25 A.U./ml, control and thrombin-challenged, respectively), indicating increased monolayer permeability in thrombin-challenged cells. Atorvastatin pretreatment significantly preserved endothelial junction integrity after thrombin challenge (bottom chamber FITC-Dextran fluorescent density  = 3.0±0.64 A.U./ml), which was reversed by co-pretreatment with 100 µM MVA or 10 µM GGPP, and partially by10 µM FPP (Figure 4B, FITC-dextran fluorescent density in bottom chamber  = 10.3±1.1, 10.1±1.0 and 5.7±1.9 A.U./ml for MVA, GGPP, and FPP, respectively ).

We next examined VE-cadherin targeting in HUVECS, to further confirm that inhibiting small GTPase translocation with atorvastatin affects endothelial cell integrity. In untreated controls, VE-cadherin staining shows random and loose junctions between endothelial cells. Large intracellular gaps and VE-cadherin junction dissociation were observed in HUVECs challenged with thrombin (Figure 4C top panel). Pretreatment with atorvastatin increased VE-cadherin targeting to junctions, and preserved these junctions after thrombin challenge (Figure 4C second panel). The effect of atorvastatin on VE-cadherin targeting was partially attenuated by 100 µM MVA, 10 µM GGPP, but not by10 µM FPP (Figure 4C third, bottom and fourth panel).

### Rho and Rac, but not Cdc42, regulate endothelial cell barrier integrity

**Figure 5:**
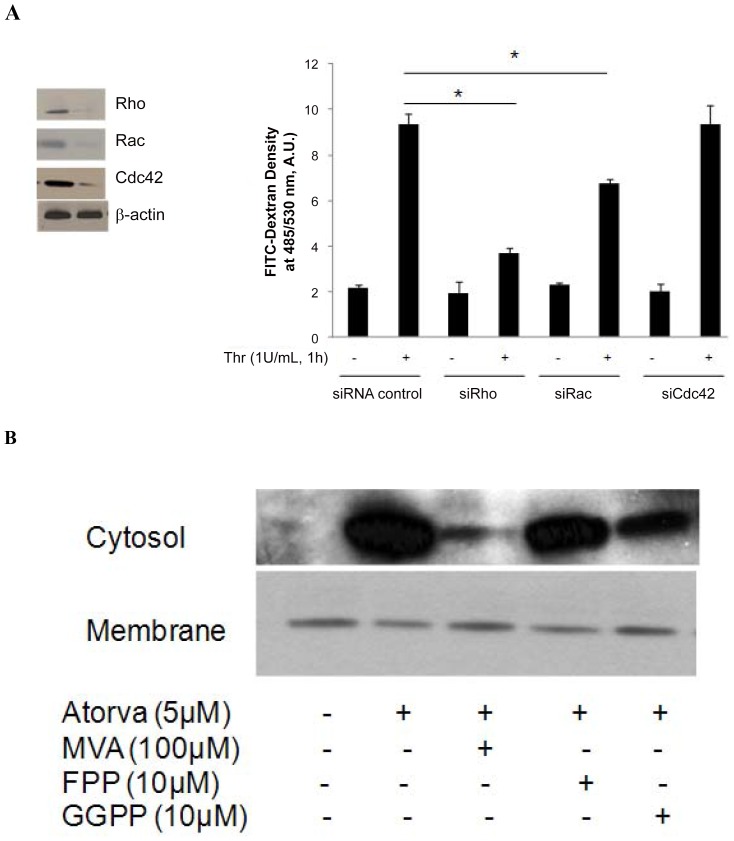
Rho and Rac regulate endothelial junction integrity. (A) RhoA, Rac1 or Cdc42 were silenced in HUVECs using 100 nM siRNA for 72 hours, and the cell lysates analyzed by chemiluminescence Western blot, as described in Methods. For the transwell experiments, proteins were silenced as indicated, and permeability tests performed. Monolayer permeability is determined by FITC-dextran fluorescence in the bottom chamber. Data are expressed as means ± S.E. (*n*≥4 samples per group), * *p*<0.05. (B) HUVECs treated with atorvastatin in the presence of MVA, FPP or GGPP as indicated. After isolating cell membrane and cytosolic fractions, the GTP-binding Rho is determined by pull-down kit and by Western blotting as described in Methods.

Permeability assays and VE-cadherin staining clearly showed that small GTPase geranylgeranylation regulates endothelial junction integrity. To identify the small GTPase proteins involved in maintaining extracellular junctions, we silenced Rho, Rac, and Cdc42 individually in HUVECs. Endothelial barrier integrity was assayed in control and small GTPase silenced HUVECs by FITC-dextran leakage, following a 1 hour thrombin challenge. Rho silencing reduced cellular junction leakage 61% relative to controls, whereas Rac silencing reduced leakage by 22% (3.7±0.2, 6.7±0.15, and 9.3±0.47 A.U./ml, Rho, Rac and control siRNA, respectively). In contrast, Cdc42 silencing did not change endothelial barrier function compared to controls (9.36±0.82 vs. 9.3±0.47 A.U./ml, figure 5A).

We next determined whether GTP remains associated with Rho during its translocation between the cell membrane and cytosol. Analysis of isolated cell membrane and cytosolic fractions showed that Rho GTP loading is preserved during translocation between membrane and cytosol after atorvastatin treatment in the presence of MVA, GGPP or FPP (figure 5B).

### Small GTPase geranylgeranylation regulates endothelial junction integrity

**Figure 6:**
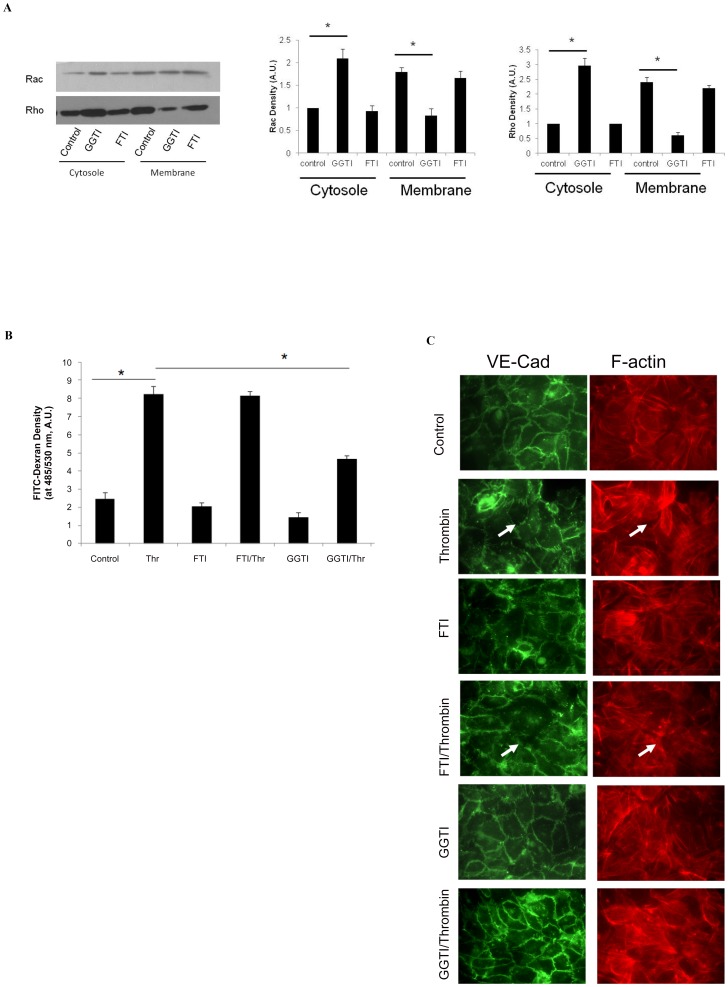
Geranylgeranylation of small GTPases is critical for endothelial junction integrity. (A) HUVECs were treated with 100 µM GGTI overnight, and cytosolic and membrane protein fractions isolated. (B) HUVECs in transwell experiments were treated with 100 µM GGTI or 100 µM FTI overnight, followed by 1 U/ml thrombin challenge. Monolayer permeability is determined by FITC-dextran fluorescent density in the bottom chamber medium. Data are expressed as means ± S.E. (*n* = 4 samples per group), * *p*<0.05. (C) HUVECs seeded on glass slides were treated with GGTI or FTI, followed by thrombin challenge as indicated. Slides were stained with VE-cadherin primary antibody and Texas Red-conjugated secondary antibody, and then visualized by fluorescence microscopy as described in Methods. The white arrows indicate gaps between endothelial cells.

We finally investigated the role of geranylgeranylation of small GTPases in cellular junction integrity, using the pharmacological agents GGTI and FTI, inhibitors of protein geranylgeranylation and farnesylation, respectively. Analysis of isolated cellular membrane and cytosolic fractions from HUVECs pretreated with GGTI showed an increase in cytosolic Rho and a decrease in membrane Rho. GGTI also had a similar effect on Rac (figure 6 A). In contrast, pretreatment with FTI had no effect on Rho or Rac distribution to membrane and cytosolic fractions. To confirm that inhibiting geranylgeranylation of small GTPases using GGTI alters cellular junction integrity, we measured HUVEC monolayer permeability by FITC-Dextran leakage in thrombin-challenged cells in the presence of GGTI or FTI. Compared to thrombin-challenge alone, pre-treatment with GGTI, but not FTI, significantly reduced HUVEC permeability after thrombin challenge (8.23±0.45, 4.67±0.15, and 8.13±0.25 A.U./ml FITC-Dextran in bottom chamber, control, GGTI, and FTI, respectively). Consistent with the results of permeability assays, immunocytochemistry showed that FTI treatment did not alter VE-cadherin targeting compared to controls, both at rest and after thrombin challenge. In contrast, GGTI treatment dramatically increased VE-cadherin targeting to junctions in both resting and thrombin challenged cells (Figure 6 C).

## Discussion

In this study, for the first time, we clarify the importance of small GTPase protein isoprenylation in processes regulating cellular junction integrity, and provide insight into the mechanism of statin-mediated anti-inflammatory effects. We also identify the temporal relationship between small GTPase GTP loading and isoprenylation. Finally, we determine the relative importance of Rho, Rac and Cdc42 in regulating endothelial junction integrity.

There are two preconditions for small GTPase protein function. The first is GTP loading, the exchange of bound GDP for GTP, which increases their activity [11]. Second, post-translational isoprenylation mediates small GTPase protein binding to the cell membrane [23], where they can regulate MLCK activity, which subsequently controls many functions, including cell shape, lamellipodia formation and junctional complex formation. Current opinion is that GTP loading is critical for small GTPase function and has been widely investigated [11], [24]. In the current study, atorvastatin strongly increased small Rho GTPases GTP loading. Concurrently, atorvastatin significantly inhibited small GTPases geranylgeranylation and caused a series of endothelial cell structural and functional changes. These two separate but important small GTPases modifications offer new opportunities to recognize the importance of both GTP loading and isoprenylation for endothelial cell junction formation and integrity. Consistent with previous reports, our results suggest that increased GTP loading of small GTPases following atorvastatin treatment may be a consequence of small GTPase dissociation with a negative regulator, the Rho guanine nucleotide dissociation inhibitor (Rho GDI) [25] or by inhibition of another factor, GAP. However, the significance of increased GTP loading, as well as the detailed mechanisms producing it, remains unknown.

In addition to GTP loading, accumulating evidence indicates that lipidation of small GTPase proteins, geranylgeranylation for Rho, Rac and Cdc42, and farnesylation for Ras is also crucial for cellular junction formation and maintaining cell shape [26], [27]. Statins inhibit synthesis of short-chain isoprenoids such as FPP and GGPP, thereby blocking small GTPase lipidation. Unmodified small GTPases do not have cellular membrane affinity, loss of which causes numerous down-stream effects, including blunting of NAPDH oxidase activity(via Rac) [3], cellular cytoskeleton rearrangement (via Rho) [28] and inhibition of cellular lamellipodia formation (via Cdc42) [29]. Our current study showed that atorvastatin inhibits Rho translocation to the endothelial cell membrane, altering cytoskeletal rearrangement response to stimuli, including thrombin challenge. Similarly, the geranylgeranylation inhibitor GGTI, but not the farnesylation inhibitor FTI, inhibited Rho translocation to the cell membrane, thereby reducing cytoskeleton stress fiber formation and preserving endothelial barrier integrity.

The detailed mechanism by which atorvastatin preserves endothelial junction integrity is not completely defined. Previous studies have shown that statins induce expression of PECAM1and integrin β4, critical components of endothelial junctions,and eNOS, which is involved in endothelial inflammatory responses [16], [26], [30], [31]. However, these findings do not account for the fundamental changes to cell junction structure, or the inhibition of stress fiber formation, observed with statin treatment [32], [33]. Our results strongly suggest that small GTPase isoprenylation, rather than GTP-loading, plays an important role in atorvastatin-mediated inhibition of stress fiber formation and preservation of cellular junction integrity.

In conclusion, our study clearly confirms the importance of post-translational isoprenylation of small GTPases to normal endothelium function, including maintenance of barrier integrity and endothelial inflammatory responses. The current study also determined the importance of Rho and Rac but not Cdc42, in mediating statin’s effects on endothelial barrier integrity and inflammatory responses. The ability of statins to inhibit isoprenylation has given us greater insight into the role of small GTPases in endothelial cell function.

## References

[1] Cordle A, Landreth G (2005) 3-Hydroxy-3-methylglutaryl-coenzyme A reductase inhibitors attenuate beta-amyloid-induced microglial inflammatory responses. J Neurosci 25: 299–307. 15647473 10.1523/JNEUROSCI.2544-04.2005 PMC6725473

[2] Vecchione C, Brandes RP (2002) Withdrawal of 3-hydroxy-3-methylglutaryl coenzyme A reductase inhibitors elicits oxidative stress and induces endothelial dysfunction in mice. Circ Res 91: 173–9. 12142351 10.1161/01.res.0000028004.76218.b8

[3] Chen W, Pendyala S, Natarajan V, Garcia JG, Jacobson JR (2008) Endothelial cell barrier protection by simvastatin: GTPase regulation and NADPH oxidase inhibition. Am J Physiol Lung Cell Mol Physiol 295: L575–83. 18658277 10.1152/ajplung.00428.2007 PMC2575942

[4] Morofuji Y, Nakagawa S, So G, Hiu T, Horai S, et al (2010) Pitavastatin strengthens the barrier integrity in primary cultures of rat brain endothelial cells. Cell Mol Neurobiol 30: 727–35. 20127168 10.1007/s10571-010-9497-9 PMC11498758

[5] Jacobson JR, Barnard JW, Grigoryev DN, Ma SF, Tuder RM, et al (2005) Simvastatin attenuates vascular leak and inflammation in murine inflammatory lung injury. Am J Physiol Lung Cell Mol Physiol 288: L1026–32. 15665042 10.1152/ajplung.00354.2004

[6] Spindler V, Schlegel N, Waschke J ( 2010) Role of GTPases in control of microvascular permeability. Cardiovasc Res 87: 243–53. 20299335 10.1093/cvr/cvq086

[7] Wojciak-Stothard B, Potempa S, Eichholtz T, Ridley AJ (2001) Rho and Rac but not Cdc42 regulate endothelial cell permeability. J Cell Sci 114: 1343–55. 11257000 10.1242/jcs.114.7.1343

[8] Wojciak-Stothard B, Ridley AJ (2002) Rho GTPases and the regulation of endothelial permeability. Vascul Pharmacol 39: 187–99. 12747959 10.1016/s1537-1891(03)00008-9

[9] Dudek SM, Birukov KG, Zhan X, Garcia JG (2002) Novel interaction of cortactin with endothelial cell myosin light chain kinase. Biochem Biophys Res Commun 298: 511–9. 12408982 10.1016/s0006-291x(02)02492-0

[10] Zhao D, Pothoulakis C (2003) Rho GTPases as therapeutic targets for the treatment of inflammatory diseases. Expert Opin Ther Targets 7: 583–92. 14498821 10.1517/14728222.7.5.583

[11] Sugden PH, Clerk A (2000) Activation of the small GTP-binding protein Ras in the heart by hypertrophic agonists. Trends Cardiovasc Med 10: 1–8. 11150721 10.1016/s1050-1738(00)00038-4

[12] McTaggart SJ (2006) Isoprenylated proteins. Cell Mol Life Sci 63: 255–67. 16378247 10.1007/s00018-005-5298-6 PMC11136304

[13] Hooff GP, Wood WG, Muller WE, Eckert GP (2010) Isoprenoids, small GTPases and Alzheimer's disease. Biochim Biophys Acta 1801: 896–905. 20382260 10.1016/j.bbalip.2010.03.014 PMC2886181

[14] Pechlivanis M, Kuhlmann J (2006) Hydrophobic modifications of Ras proteins by isoprenoid groups and fatty acids – More than just membrane anchoring. Biochim Biophys Acta 1764: 1914–31. 17110180 10.1016/j.bbapap.2006.09.017

[15] Ishibashi T (2011) [Anti-atherosclerotic and anti-inflammatory effects of statin on cardiovascular disease and their mechanisms]. Nihon Rinsho 69: 79–84. 21226265

[16] Jacobson JR, Dudek SM, Birukov KG, Ye SQ, Grigoryev DN, et al (2004) Cytoskeletal activation and altered gene expression in endothelial barrier regulation by simvastatin. Am J Respir Cell Mol Biol 30: 662–70. 14630613 10.1165/rcmb.2003-0267OC

[17] Ramos JW (2008) The regulation of extracellular signal-regulated kinase (ERK) in mammalian cells. Int J Biochem Cell Biol 40: 2707–19. 18562239 10.1016/j.biocel.2008.04.009

[18] Park HJ, Kong D, Iruela-Arispe L, Begley U, Tang D, et al (2002) 3-hydroxy-3-methylglutaryl coenzyme A reductase inhibitors interfere with angiogenesis by inhibiting the geranylgeranylation of RhoA. Circ Res 91: 143–50. 12142347 10.1161/01.res.0000028149.15986.4c

[19] Xu H, Zeng L, Peng H, Chen S, Jones J, et al (2006) HMG-CoA reductase inhibitor simvastatin mitigates VEGF-induced "inside-out" signaling to extracellular matrix by preventing RhoA activation. Am J Physiol Renal Physiol 291: F995–1004. 16774905 10.1152/ajprenal.00092.2006

[20] Herwald H, Dedio J, Kellner R, Loos M, Muller-Esterl W (1996) Isolation and characterization of the kininogen-binding protein p33 from endothelial cells. Identity with the gC1q receptor. J Biol Chem 271: 13040–7. 8662673 10.1074/jbc.271.22.13040

[21] Birukova AA, Chatchavalvanich S, Rios A, Kawkitinarong K, Garcia JG, et al (2006) Differential regulation of pulmonary endothelial monolayer integrity by varying degrees of cyclic stretch. Am J Pathol 168: 1749–61. 16651639 10.2353/ajpath.2006.050431 PMC1606576

[22] Shivanna M, Jalimarada SS, Srinivas SP (2010) Lovastatin inhibits the thrombin-induced loss of barrier integrity in bovine corneal endothelium. J Ocul Pharmacol Ther 26: 1–10. 20148651 10.1089/jop.2009.0025 PMC3096541

[23] Kowluru RA, Kanwar M (2009) Translocation of H-Ras and its implications in the development of diabetic retinopathy. Biochem Biophys Res Commun 387: 461–6. 19607814 10.1016/j.bbrc.2009.07.038 PMC2744948

[24] Nassar N, Cancelas J, Zheng J, Williams DA, Zheng Y (2006) Structure-function based design of small molecule inhibitors targeting Rho family GTPases. Curr Top Med Chem 6: 1109–16. 16842149 10.2174/156802606777812095

[25] Cordle A, Koenigsknecht-Talboo J, Wilkinson B, Limpert A, Landreth G (2005) Mechanisms of statin-mediated inhibition of small G-protein function. J Biol Chem 280: 34202–9. 16085653 10.1074/jbc.M505268200

[26] Wei H, Fang L, Song J, Chatterjee S (2005) Statin-inhibited endothelial permeability could be associated with its effect on PECAM-1 in endothelial cells. FEBS Lett 579: 1272–8. 15710425 10.1016/j.febslet.2005.01.020

[27] Tafazoli F, Magnusson KE, Zheng L (2003) Disruption of epithelial barrier integrity by Salmonella enterica serovar typhimurium requires geranylgeranylated proteins. Infect Immun 71: 872–81. 12540569 10.1128/IAI.71.2.872-881.2003 PMC145360

[28] Kato T, Hashikabe H, Iwata C, Akimoto K, Hattori Y (2004) Statin blocks Rho/Rho-kinase signalling and disrupts the actin cytoskeleton: relationship to enhancement of LPS-mediated nitric oxide synthesis in vascular smooth muscle cells. Biochim Biophys Acta 1689: 267–72. 15276654 10.1016/j.bbadis.2004.04.006

[29] Burnham CA, Shokoples SE, Tyrrell GJ (2007) Rac1, RhoA, and Cdc42 participate in HeLa cell invasion by group B streptococcus. FEMS Microbiol Lett 272: 8–14. 17517067 10.1111/j.1574-6968.2007.00768.x

[30] Chen W, Garcia JG, Jacobson JR (2010) Integrin beta4 attenuates SHP-2 and MAPK signaling and reduces human lung endothelial inflammatory responses. J Cell Biochem 110: 718–24. 20512931 10.1002/jcb.22582 PMC2879705

[31] Mital S, Zhang X, Zhao G, Bernstein RD, Smith CJ, et al (2000) Simvastatin upregulates coronary vascular endothelial nitric oxide production in conscious dogs. Am J Physiol Heart Circ Physiol 279: H2649–57. 11087217 10.1152/ajpheart.2000.279.6.H2649

[32] Horn MP, Knecht SM, Rushing FL, Birdsong J, Siddall CP, et al (2008) Simvastatin inhibits Staphylococcus aureus host cell invasion through modulation of isoprenoid intermediates. J Pharmacol Exp Ther 326: 135–43. 18388257 10.1124/jpet.108.137927 PMC4482125

[33] Bacova B, Radosinska J, Knezl V, Kolenova L, Weismann P, et al (2010) Omega-3 fatty acids and atorvastatin suppress ventricular fibrillation inducibility in hypertriglyceridemic rat hearts: implication of intracellular coupling protein, connexin-43. J Physiol Pharmacol 61: 717–23. 21224503

